# Effectiveness of Ebola Treatment Units and Community Care Centers — Liberia, September 23–October 31, 2014

**Published:** 2015-01-30

**Authors:** Michael L. Washington, Martin L. Meltzer

**Affiliations:** 1 Division of Preparedness and Emerging Infections, National Center for Emerging and Zoonotic Infectious Diseases, CDC

Previous reports have shown that an Ebola outbreak can be slowed, and eventually stopped, by placing Ebola patients into settings where there is reduced risk for onward Ebola transmission, such as Ebola treatment units (ETUs) and community care centers (CCCs) or equivalent community settings that encourage changes in human behaviors to reduce transmission risk, such as making burials safe and reducing contact with Ebola patients (1,2). Using cumulative case count data from Liberia up to August 28, 2014, the EbolaResponse model (3) previously estimated that without any additional interventions or further changes in human behavior, there would have been approximately 23,000 reported Ebola cases by October 31, 2014. In actuality, there were 6,525 reported cases by that date. To estimate the effectiveness of ETUs and CCCs or equivalent community settings in preventing greater Ebola transmission, CDC applied the EbolaResponse model (3) to the period September 23–October 31, 2014, in Liberia. The results showed that admitting Ebola patients to ETUs alone prevented an estimated 2,244 Ebola cases. Having patients receive care in CCCs or equivalent community settings with a reduced risk for Ebola transmission prevented an estimated 4,487 cases. Having patients receive care in either ETUs or CCCs or in equivalent community settings, prevented an estimated 9,100 cases, apparently as the result of a synergistic effect in which the impact of the combined interventions was greater than the sum of the two interventions. Caring for patients in ETUs, CCCs, or in equivalent community settings with reduced risk for transmission can be important components of a successful public health response to an Ebola epidemic.

One component of the national strategy in Liberia for responding to the ongoing Ebola epidemic is to isolate persons with suspected, probable, or confirmed Ebola in ETUs or, when ETUs are full or otherwise not available, in community-based settings such as CCCs, where there also is a reduced risk for Ebola transmission (4). The EbolaResponse model was used to estimate how many Ebola cases were averted in Liberia during September 23–October 31, 2014, because of the use of ETUs, CCCs, and equivalent community settings. This period was selected for study because there was a notable increase in interventions during that period that correlated with a decrease in cases (4,5).

The spreadsheet-based EbolaResponse modeling tool tracks patients through the following states of Ebola virus infection and disease: susceptible to disease, infected, incubating, infectious, and recovered. Data from reports of previous Ebola outbreaks were used to model the daily change of patients’ status between the disease states. For example, a probability distribution to characterize the likelihood of incubating a given number of days was built using previously published data (3). Patients in the modeled population were distributed into three categories: 1) hospitalized in an ETU; 2) placed into a CCC or a home in a community setting where there was a reduced risk for disease transmission and an emphasis on changing human behaviors with regard to safe burials and reducing contact with patients; and 3) left at home with no effective isolation or safe burials. Both the risk for onward disease transmission by patient category and the percentage of patients in each category were calculated by altering these values until the estimates of cumulative cases over time produced by the model (the model “fit” [3]) closely matched those of the actual data.

An initial estimate of cumulative cases was made by fitting the EbolaResponse model to cumulative Liberian case count data (i.e., confirmed, probable, and suspected cases) from March 27 to November 15, 2014 (6). A good fit of the estimated cases to actual cases was obtained when patients were distributed, for the period September 23–October 31, 2014, into the three categories as follows: 20% of Ebola cases in ETUs, 35% in CCCs or equivalent community settings with a reduced risk for Ebola transmission, and 45% at home without effective isolation or safe burials. Three scenarios were then built to estimate the impact of ETUs and CCCs during the study period.

## Three Estimation Scenarios

In scenario 1, to estimate the impact of placing Ebola patients in ETUs, for the period September 23–October 31, 2014, the 20% of all Ebola patients calculated to be in ETUs were moved to the category of patients who were at home without effective isolation or safe burials. The 35% of patients calculated to be in CCCs or equivalent community settings with reduced risk were unchanged. The model was refitted to produce estimates of cases that would have occurred without any patients in ETUs.

In scenario 2, to estimate the impact of the 35% of Ebola patients calculated to be in CCCs or equivalent community settings with reduced risk for Ebola, the 35% were moved to the category of patients who were at home without effective isolation or safe burials. The 20% of patients in ETUs were unchanged, and the model was refitted to provide estimates of cases that would have occurred without any patients in CCCs or equivalent community settings.

In scenario 3, to measure the impact of placing patients in either ETUs or CCCs, the 55% of patients calculated to be in either ETUs or CCCs or equivalent community settings were moved to the category of patients who were at home without effective isolation or safe burials. The model was then refitted to provide estimates of cases without any patients in either ETUs or CCCs (Table 1).

## Number of Ebola Cases Averted

The cumulative number of estimated cases during March 27–October 31, 2014, based on model assumptions, was 6,218, compared with 6,525 cumulative cases reported in Liberia (6). If no patients had been hospitalized in ETUs starting on September 23, 2014, (scenario 1), there would have been an estimated additional 2,244 cases by October 31, 2014 (Figure, Table 2). If no patients had been placed into CCCs or equivalent community settings with reduced risk for transmission, there would have been an estimated additional 4,487 cases by October 31, 2014. If no patients were placed into either ETUs or CCCs or the equivalent settings with reduced risk for Ebola transmission (scenario 3), there would have been an estimated additional 9,097 cases by October 31, 2014 (Figure).

Also estimated were the number of Ebola cases that would be averted for the period September 23–October 31, 2014, by placing only 1% of patients in either an ETU or a CCC or both. This calculation assumed that that the number of cases averted per 1% of patients placed into ETUs or CCCs did not change as the total percentage of patients in these care settings increased (i.e., a linear correlation was assumed between cases averted and percentage of patients in the care settings).

During September 23–October 31, 2014, for every 1% of patients placed into ETUs, an estimated 112 cases would have been averted (Table 2). Similarly, for every 1% of patients placed into CCCs or equivalent settings with reduced risk for transmission, an estimated 128 cases would have been averted. For every 1% increase in patients placed into ETUs or CCCs or equivalent settings, an estimated 165 cases would have been averted (Table 2).

Also calculated were the numbers of days required in each scenario for the number of cases to double (doubling time). For the study period, under scenario 1 (no ETUs operating) and scenario 2 (no CCCs or equivalent settings), cases doubled in 23 and 20 days, respectively. Under scenario 3 (neither ETUs nor CCCs operating), cases doubled in 18 days.

### Discussion

During September 23–October 31, 2014, placing Ebola patients into ETUs or CCCs or equivalent settings with reduced transmission risk prevented an estimated 9,097 cases of Ebola in Liberia. The findings in this report support those from an earlier report on Lofa County, Liberia, that found ETUs played a major role in reducing the number of cases in October (5).

Of note is the finding that scenario 3 (combined effect of ETUs and CCCs) resulted in more cases averted than the sum of the estimated cases averted from scenario 1 (patients in ETUs) and scenario 2 (patients in CCCs and equivalent community settings). This apparent synergistic effect from having both ETUs and CCCs operating in a community during an Ebola epidemic might have resulted from the alteration of the doubling time.

The findings in this report are subject to at least two limitations. First, the findings are limited by the previously described limitations associated with using the EbolaResponse model (3). Second, the study is limited by the implicit assumption of a constant relationship (i.e., linear correlation) between patients in ETUs or CCCs and cases averted. In reality, such relationships most likely vary with changes in the number of total cases and the number of patients in ETUs or CCCs. Thus, caution should be exercised when using these results to estimate the potential impact of ETUs and CCCs in other settings.

The results of this study demonstrate the importance of effective isolation of Ebola patients in ETUs and CCCs in controlling an Ebola outbreak. At the peak of an Ebola outbreak in a community, there might be insufficient ETU capacity to accommodate all Ebola patients (4). Under such circumstances, provision of CCCs and community-based programs that encourage safe burials and reduced contact with Ebola patients should be established at least as interim measures until adequate treatment capacity is available. These data indicate that the rapid initiation of a multifaceted response to a large Ebola outbreak in Liberia was warranted.

What is already known on this topic?

Previous studies have documented the decline in the number of Ebola cases in the Liberian counties of Montserrado and Lofa resulting from public health interventions. These measures included the establishment of Ebola treatment centers (ETUs) and community care centers (CCCs) and the provision of community-based education to encourage changes in human behaviors, such as providing safe burials and reducing contact with patients.

What is added by this report?

This report provides estimates of the relative impact ETUs and CCCs and equivalent community settings with reduced risk for Ebola transmission. The findings indicate that during September 23–October 31, 2014, hospitalizing approximately 20% of all Ebola patients in ETUs prevented an estimated 2,244 cases, and placing 35% of patients in CCCs or equivalent community settings that encourage safe burials and reduced contact with patients prevented an estimated 4,487 cases. Together, these interventions prevented an estimated 9,097 cases; the impact of the combined interventions was greater than the sum of the individual interventions.

What are the implications for public health practice?

These data demonstrate that, when responding to large-scale outbreaks of Ebola, rapid initiation of both ETUs and CCCs can avert cases of Ebola.

## Figures and Tables

**FIGURE:**
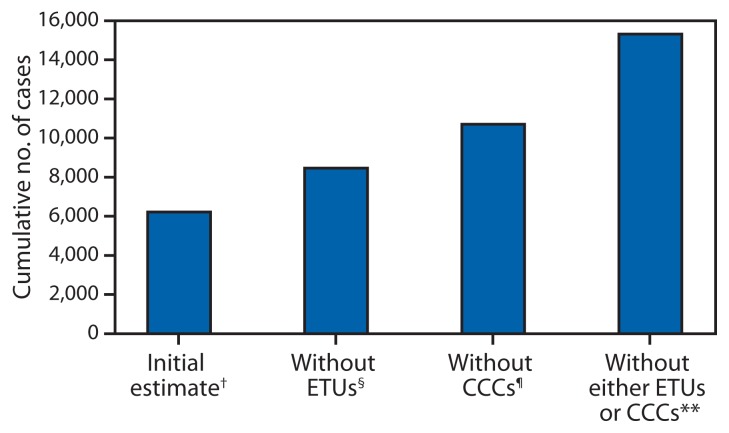
Estimates of the cumulative number of Ebola cases with and without Ebola treatment units (ETUs) and community care centers (CCCs)* — Liberia, September 23–October 31, 2014 * CCCs or equivalent community settings with a reduced risk for Ebola transmission (including safe burial and community-based programs to change human behavior to reduce contact with patients). ^†^ The initial estimate was calculated by fitting the EbolaResponse model to cumulative cases in Liberia for the period March 27–November 15, 2014. From this fit, 6,218 cumulative cases were estimated to have occurred by October 31, 2014. During September 23–October 31, 2014, it was calculated that 20% of Ebola patients were in ETUs, 35% were in CCCs or equivalent community settings with a reduced risk for Ebola transmission (including safe burial), and, 45% were at home without effective isolation, resulting in an increased risk for Ebola transmission (including unsafe burials). ^§^ The impact if there were no ETUs was calculated by moving the 20% of Ebola patients in ETUs in the initial estimate to the category of patients who were at home without effective isolation (including unsafe burials). ^¶^ The impact if there were no CCCs, safe burials, and other community-based interventions to reduce the risk for transmission was calculated by moving the 35% of patients in CCCs or equivalent community settings to the category of patients who were at home without effective isolation (including unsafe burials). ** The combined impact if there were no ETUs and CCCs, safe burials and other community-based interventions to reduce the risk for transmission was calculated by moving both the 20% of patients in ETUs and 35% of patients in CCCs or equivalent community settings to the category of patients who were at home without effective isolation (including unsafe burials).

**TABLE 1:** Percentage of Ebola cases in each category of patient care, by three scenarios used to estimate the impact if there were no Ebola treatment units (ETUs) and community care centers (CCCs)* — Liberia, September 23–October 31, 2014

Patient care category	Initial estimates of % of patients by category†	% estimates if no ETUs (scenario 1)	% estimates if no CCCs (scenario 2)	% estimates if no ETUs or CCCs (scenario 3)
ETUs	20	0	20	0
CCCs	35	35	0	0
At home without effective isolation§	45	65	80	100

* CCCs or equivalent community settings with a reduced risk for Ebola transmission (including safe burial and community-based programs to change human behavior to reduce contact with patients).

† The initial estimates were calculated by fitting the EbolaResponse model to cumulative cases in Liberia for the period March 27–November 15, 2014. From this fit, 6,218 cumulative cases were estimated to have occurred by October 31, 2014. During September 23–October 31, 2014, it was calculated that 20% of Ebola patients were in ETUs, 35% were in CCCs or equivalent community settings with a reduced risk for Ebola transmission (including safe burial), and, 45% were at home without effective isolation, resulting in an increased risk for Ebola transmission (including unsafe burials).

§ Resulting in an increased risk for Ebola transmission (including unsafe burials).

**TABLE 2:** Estimated number of Ebola cases averted per 1% change in the number of patients in Ebola treatment units (ETUs) and community care centers (CCCs)* — Liberia, September 23–October 31, 2014

Patient care category	No. of cases averted	No. of cases averted per 1% change in patients†
ETUs	2,244	112
CCCs	4,487	128
Patients in either ETUs or CCCs	9,097	165

* CCCs or equivalent community settings with a reduced risk for Ebola transmission (including safe burial and community-based programs to change human behavior to reduce contact with patients).

† For every 1% of patients placed into the relevant patient care category (ETUs, CCCs, or either), the number of cases that would be averted (assuming a linear correlation between cases averted and patients in ETUs or CCCs or either).
